# Supplementary figures and images for: Uniparental Inheritance and Recombination as Strategies to Avoid Competition and Combat Muller’s Ratchet among Mitochondria in Natural Populations of the Fungus Amanita phalloides
Source: J Fungi (Basel). 2023 Apr 15;9(4):476. doi: 10.3390/jof9040476 (PMC10142858; doi:10.3390/jof9040476)

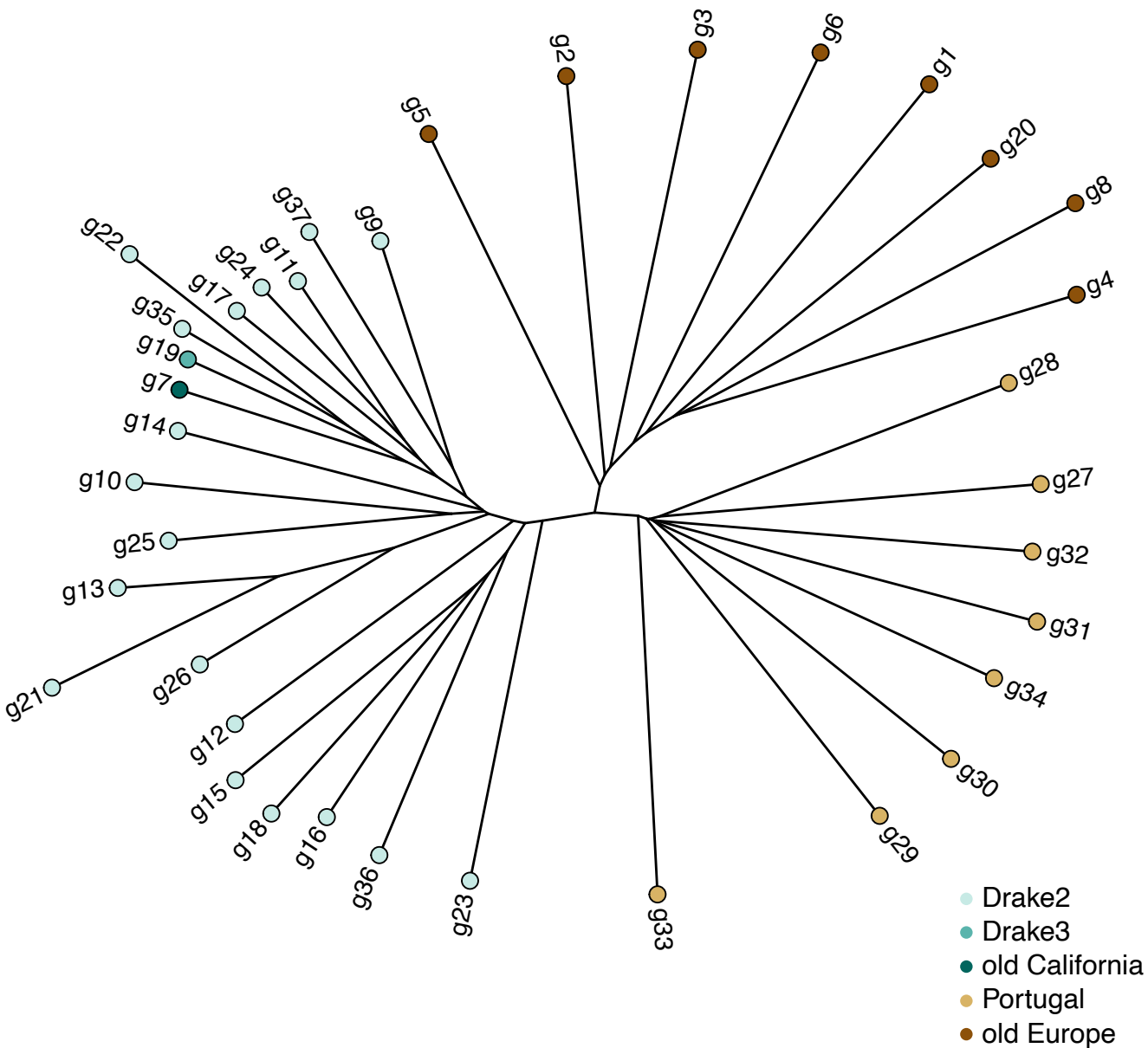

Supplement: Supplementary file 1 [file jof-09-00476-s001.zip › Supplementary Figure S1.pdf]

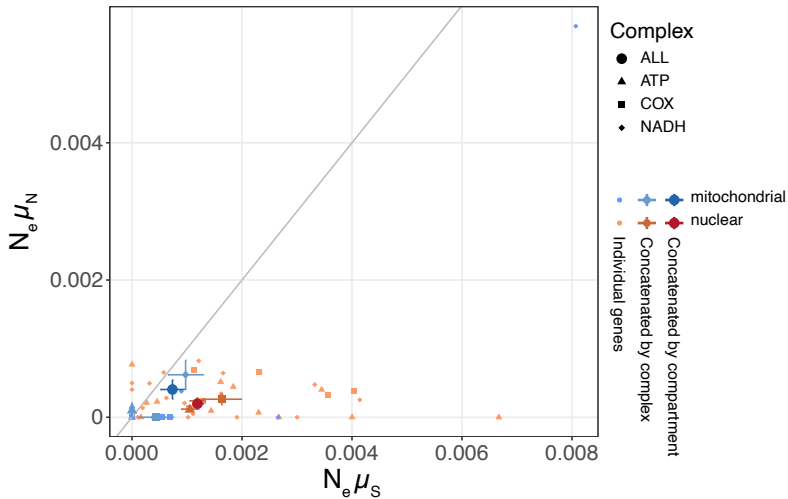

Supplement: Supplementary file 1 [file jof-09-00476-s001.zip › Supplementary Figure S2.pdf]
